# Associations of Physical Activity with One-Year Functional and Cognitive Trajectories Following High-Speed Resistance Training in Older Adults

**DOI:** 10.3390/sports14090386

**Published:** 2026-09-02

**Authors:** Alexandre Duarte Martins, João Paulo Brito, Nuno Batalha, Bruno Gonçalves, Rafael Oliveira, Orlando Fernandes

**Affiliations:** 1 Comprehensive Health Research Centre (CHRC), Escola de Saúde e Desenvolvimento Humano, Departamento de Desporto e Saúde, Universidade de Évora, Largo dos Colegiais 2, 7004-516 Évora, Portugal; nmpba@uevora.pt (N.B.); bgoncalves@uevora.pt (B.G.); orlandoj@uevora.pt (O.F.); 2 Life Quality Research Center (CIEQV), Santarém Polytechnic University, Complexo Andaluz, Apartado 279, 2001-904 Santarém, Portugal; jbrito@esdrm.ipsantarem.pt (J.P.B.); rafaeloliveira@esdrm.ipsantarem.pt (R.O.); 3 School of Sport of Rio Maior, Santarém Polytechnic University, Av. Dr. Mário Soares, 2040-413 Rio Maior, Portugal; 4 Research Center in Sport Sciences, Health Sciences and Human Development (CIDESD), Santarém Polytechnic University, Av. Dr. Mário Soares, 2040-413 Rio Maior, Portugal; 5 Healthy-Age Research Network: Active Aging, Exercise and Health, Consejo Superior de Deportes (CSD), Ministry of Culture and Sport of Spain, 28040 Madrid, Spain

**Keywords:** velocity-based training, aging, physical activity, activities of daily living, cognition

## Abstract

Background: Engaging in regular physical activity (PA) after completing structured exercise programmes may contribute to sustaining functional and cognitive gains in older adults over the long term. This study investigated whether different levels of PA during the one-year follow-up were associated with the preservation of the physical and cognitive gains achieved after a high-speed resistance training (HSRT) intervention lasting 16 weeks. Methods: Thirty-six community-dwelling older adults who had previously finished a supervised 16-week HSRT programme were monitored for one year. According to self-reported PA assessed at the one-year follow-up, participants were allocated to either a light activity group (LAG, *n* = 20) or a moderate-to-vigorous activity group (MVAG, *n* = 16). The following tests were used to assess physical function: six-minute walk test (6MWT), chair-stand test, seated medicine ball throw (SMBT), timed up-and-go test (TUG), and handgrip strength (HGS), whereas global cognitive function was assessed with the Mini-Mental State Examination (MMSE). Results: Exploratory between-group comparisons indicated that participants in the MVAG tended to show better chair-stand performance at the six-month (*d_unb_* = 0.76 [0.09 to 1.45]) and one-year (*d_unb_* = 1.02 [0.34 to 1.74]) assessments. SMBT performance declined similarly in both groups throughout follow-up (LAG: *d_unb_* = −0.41 [−0.61 to −0.24]; MVAG: *d_unb_* = −0.44 [−0.72 to −0.21]). Although the group × time interaction did not reach statistical significance (*p* = 0.079, η_p_^2^ = 0.088), exploratory analyses showed that declines in chair-stand and TUG performance occurred mainly in the LAG, pointing to possible patterns related to the maintenance of functional capacity. Cognitive performance decreased post-intervention in both groups, regardless of the PA level. Conclusions: Higher levels of self-reported PA assessed at the one-year follow-up were associated with more favourable trends in lower-limb functional performance.

## 1. Introduction

People aged 65 years and over [1] frequently exhibit age-related reductions in physical function, such as losses in muscle power, mass, and strength. These changes elevate the risk of falling and may undermine independence and autonomy [2,3]. Alongside these physical declines, aging is also accompanied by reductions in cognitive function, potentially related to changes in brain tissue volume and structure and reduced cerebral blood flow [4], which may result in slower processing speed and reduced working memory [5].

In response to these challenges, numerous studies have shown that structured exercise programmes, particularly resistance training (RT), can effectively preserve or enhance physical and cognitive capacities in older adults. Improvements have consistently been reported in functional outcomes such as the chair-stand test [6,7,8], timed up-and-go test (TUG) [8,9], and handgrip strength (HGS) [8,10], alongside favourable changes in global cognitive status measured through the Mini-Mental State Examination (MMSE) [11]. Despite the well-established benefits of conventional RT [12,13,14,15], far less attention has been directed toward high-speed resistance training (HSRT), and knowledge about its extended effects over time is still scarce [16,17,18].

HSRT emphasizes high-velocity movements during the concentric phase, followed by a slower, controlled eccentric phase, typically using low-to-moderate external loads [19]. According to Sayers et al. [19], this training modality may enhance the transfer of adaptations to real-world functional tasks. Unlike traditional RT, which primarily promotes muscle hypertrophy, HSRT predominantly induces neuromuscular adaptations [20,21,22], including increased motor unit recruitment, higher firing rates, and improved synchronization and coordination between agonist and antagonist muscles [22].

Despite the documented benefits of RT, previous studies in older adults have studied the detraining effects [6,7,8,9,10,23,24], with follow-up periods ranging from one [7] to 12 months [6,10,24]. In contrast, this study aimed to determine whether the level of habitual physical activity (PA) maintained after completing a 16-week HSRT programme influenced the long-term retention of training-related adaptations. Rather than restricting participants’ daily behaviour during follow-up, they were encouraged to remain physically active and minimise sedentary time, thereby reflecting current public health recommendations that advocate the maintenance of regular PA beyond supervised exercise programmes [25].

To operationalize habitual PA, participants were classified according to the International Physical Activity Questionnaire-Short Form (IPAQ-SF) categorical scoring protocol, which distinguishes low, moderate, and high PA levels based on validated combinations of activity frequency, duration, intensity, and weekly MET-minutes [26]. Moderate activity corresponds to ≥600 MET-min/week, while high activity requires ≥1500–3000 MET-min/week. Individuals not meeting these thresholds are classified as low active [27,28,29,30,31]. This validated categorization allows for distinguishing participants with insufficient PA from those meeting recommended PA guidelines.

Therefore, the current study sought to evaluate whether PA levels assessed at follow-up were associated with subsequent patterns of physical and cognitive function following a 16-week HSRT programme. It was hypothesized that older adults who reported higher habitual PA levels during the one-year follow-up would show long-term maintenance of the physical and cognitive gains achieved through the HSRT programme compared with those reporting lower PA levels.

## 2. Materials and Methods

### 2.1. Experimental Design

This investigation adopted a prospective longitudinal single-cohort design to evaluate whether one-year follow-up detraining period influenced the long-term preservation of the adaptations after an HSRT lasting 16 weeks. This study was conducted within the framework of the “Active Age” project, which is registered at clinicaltrials.gov (ID: NCT05586087), with its trial registration dated 19 October 2022.

The study followed the ethical guidelines of the Declaration of Helsinki and was authorised by the Institutional Ethics Committee of the host university (no. 22030). Prior to enrolment, participants were given detailed written and verbal information describing the study’s purpose, procedures, possible risks, and expected benefits. All individuals signed an informed consent form before undergoing any evaluation or participating in the intervention.

Participants completed repeated measurements at the following time points: pre-intervention (M0), post-intervention (M1), six months after the intervention (M2), and one year after the intervention (M3). The present analysis considered only the individuals assigned to the intervention group (IG). Although a control group (CG) was included during the 16-week intervention phase, it was not retained for the one-year follow-up. The institutional ethics committee advised against asking older adults to remain inactive for an extended period (intervention plus follow-up), as this would conflict with public health recommendations and raise ethical concerns. For this reason, CG participants were invited to join a different research project immediately after the intervention phase and did not undergo follow-up assessments. Consequently, only participants who completed the HSRT programme were eligible for the one-year follow-up analysis. The primary findings comparing the IG and CG during the initial 16-week intervention have been reported previously [32].

Given the modest final sample size obtained after the one-year follow-up, the present study should be considered preliminary in nature, providing exploratory evidence regarding the long-term maintenance of HSRT-induced adaptations.

The research team deliberately avoided using the term detraining, as participants were neither instructed nor encouraged to reduce their habitual PA after the intervention. Instead, consistent with ethical recommendations promoting healthy ageing, participants were advised to remain physically active and were free to continue or initiate regular exercise programmes throughout the follow-up period.

### 2.2. Participants

The intervention study enrolled 79 older adults, with 40 placed in the IG and 39 in the CG. For this longitudinal analysis, only the 36 participants from the IG who completed the M3 assessment were included. Habitual PA at the one-year follow-up was measured using the short form of the IPAQ-SF. Based on the reported PA levels, participants were subsequently classified into a moderate-to-vigorous activity group (MVAG, *n* = 16, 68.50 ± 2.09 years) or a light activity group (LAG, *n* = 20, 70.00 ± 3.66 years). Both males and females participated in the study, with sex distribution as follows: 3 males and 17 females in LAG and 3 males and 13 females in MVAG.

To strengthen methodological transparency, it was provided an excel file (Supplementary Material) adapted from Cheng [33], containing anonymized participant data across all four assessment points together with the criteria used to classify PA levels.

Eligibility criteria required participants to be at least 65 years of age, capable of independent ambulation, and able to perform activities of daily living without assistance. Individuals with diagnosed cardiovascular disease or diabetes, recent surgery (within the previous six months), or active oncological disease were excluded from participation.

Among the 79 participants enrolled, five (6.3%) did not complete the post-intervention assessment (M1) due to discomfort, loss of contact, competing exercise participation, or medical diagnosis. One additional participant was lost during the one-year follow-up due to lack of contact.

### 2.3. Assessments

All evaluations were completed over two consecutive days using standardized testing procedures. During the first assessment day, anthropometric data were collected between 08:30 and 10:30 a.m. Participants arrived after fasting for at least eight hours, having emptied their bladder, and were instructed to avoid structured exercise, alcohol, and caffeinated drinks during the previous 24 h to reduce variability.

**Figure 1:**
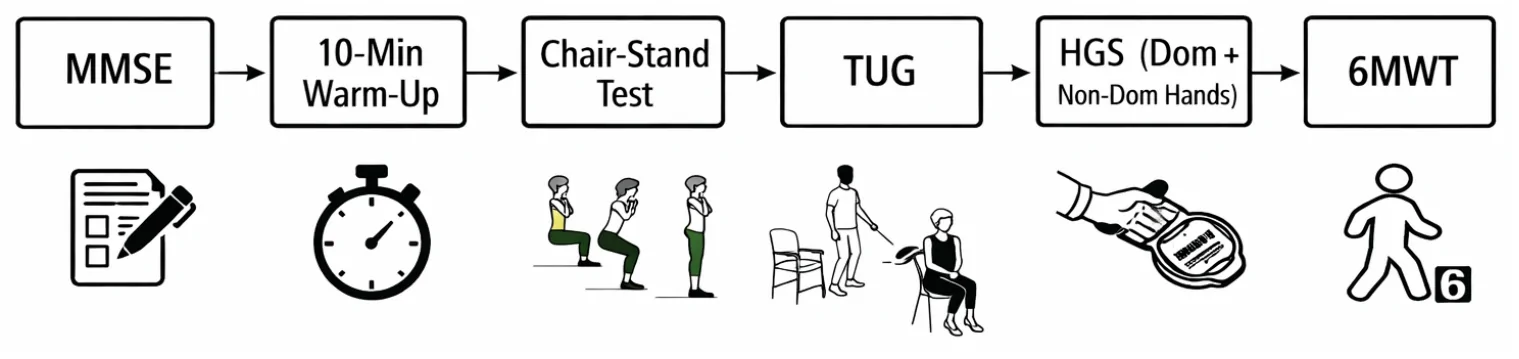
Overview of the testing sequence applied during each evaluation session.

Functional and cognitive evaluations took place the following day between 09:00 a.m. and 01:00 p.m. Participants were asked to wear appropriate sports attire to allow unrestricted movement. All tests were administered by the same experienced researcher, and the sequence was identical for every participant. The assessment battery included the MMSE, a structured 10 min warm-up, the chair-stand test, TUG, seated medicine ball throw (SMBT), HGS for both the dominant and non-dominant hands, and the six-minute walk test (6MWT) (Figure 1).

#### 2.3.1. Physical Function

Before the functional assessments, participants completed a structured 10 min warm-up that included light walking and dynamic stretching of the major muscle groups. Following the warm-up, physical function was measured by five tests. Lower-limb functional performance was evaluated with the 30 s chair-stand test [34], with movement velocity continuously captured by a validated BEAST™ inertial sensor (Beast Technologies, Brescia, Italy) [35]. Functional mobility and dynamic balance were assessed using the TUG test [34]. Upper-body muscle power was determined through the SMBT performed with a 3 kg medicine ball [36]. Maximal HGS was measured using a hydraulic hand dynamometer (JAMAR^®^, Seahan Corporation, Masan, Republic of Korea), while aerobic capacity was evaluated using the six-minute walk test (6MWT) [34].

HGS was measured on both the dominant and non-dominant hands, with the dynamometer grip individually adjusted beforehand. Participants performed the test in a standing posture, feet shoulder-width apart, arms fully extended, elbows locked at 180°, and gaze directed forward. Two maximal trials were completed for each hand, separated by one minute of rest, with each contraction maintained for at least two seconds [37]. The average of the two trials was used for statistical analysis.

#### 2.3.2. Cognitive Function

Global cognitive performance was assessed using the MMSE [38]. The Portuguese version applied in this study had been previously translated, culturally adapted, and validated by Guerreiro et al. [39]. The MMSE consists of 30 items organised into five cognitive domains: (i) orientation to time and place (10 points), (ii) immediate memory/registration (3 points), (iii) attention and calculation (5 points), (iv) delayed recall (3 points), and (v) language and visual constructive abilities (9 points), with one point specifically allocated to the visual constructive task.

#### 2.3.3. Anthropometric

Body mass and standing height were obtained using a calibrated TANITA^®^ electronic scale (MC 780MA, Amsterdam, The Netherlands) and a SECA^®^ wall-mounted stadiometer (220,Hamburg, Germany). Body mass index (BMI) was then calculated using the standard kg/m^2^ equation. All anthropometric assessments were conducted by an ISAK Level 1 certified anthropometrist to ensure methodological accuracy and consistency.

#### 2.3.4. Physical Activity

The IPAQ-SF was used to determine the participants’ PA levels [27,28,29,30,31]. The questionnaire collected information on the frequency and duration of walking, moderate-intensity activity, vigorous-intensity activity, and total physical activity, expressed as MET-minutes per week. Sedentary behaviour was also recorded by estimating sitting time on weekdays and weekend days.

Participants were subsequently classified into light, moderate, or vigorous PA categories according to the IPAQ-SF scoring criteria (Table S1, Supplementary Material). Data processing was performed using the automated scoring spreadsheet proposed by Cheng [33]. A detailed description of the self-reported activities performed by participants at the one-year follow-up is provided in Table S2.

Participants were not enrolled in any supervised RT program during the one-year follow-up. All PA was self-selected and unsupervised. As detailed in Table S2, only one participant in the MVAG reported performing structured resistance training at least twice per week. The remaining MVAG participants primarily engaged in cardiovascular-based activities, namely daily walking. In contrast, most LAG participants reported low-intensity walking, occasional outdoor activities, or no structured PA.

### 2.4. Intervention Programme

The supervised HSRT program was delivered three times weekly across a 16-week period, with training variables adjusted every two weeks to maintain progressive overload. Each session consisted of a preparatory warm-up, a central HSRT segment, and a brief recovery period. The main training segment incorporated upper- and lower-body movements, such as squats (performed either on a Smith machine or with dumbbells, depending on each participant’s functional capacity), leg press, leg extension, calf raise, seated row, pec fly, lat pulldown, and incline bench press, executed on machines (Technogym^®^, SPA, Cesena, Italy).

The HSRT protocol used in this study followed a progressive load-adjustment model based on the mean concentric velocity recorded in each exercise set. In brief, target velocity zones progressed from >1.3 m/s during weeks 1 to 4, to 1.3–1.0 m/s in weeks 5 to 10, and 1.0–0.75 m/s in weeks 11 to 16. Concentric velocity was monitored in real time using a BEAST™ inertial sensor [35]. Six sensors were paired via Bluetooth with individual mobile devices during each training session. Participants were consistently instructed to execute the concentric phase as rapidly as possible while controlling the eccentric phase for approximately 2–3 s. A full description of the training protocol, including exercise selection and weekly progression, is available in the original publication [40].

### 2.5. Statistical Analysis

All analyses were conducted in IBM SPSS Statistics (Version 26.0; IBM Corp., Armonk, NY, USA). Two-tailed tests with a significance level of *p* < 0.05 were applied. Estimation-based metrics were also reported to aid interpretation of effect magnitude and precision [41,42].

An a priori sample size calculation for the original clinical trial comparing the IG and CG during the 16-week HSRT programme was performed using G*Power (Version 3.1, Heinrich Heine University Düsseldorf, Germany) [40]. This analysis indicated that at least 79 participants were required to achieve adequate statistical power for detecting the expected intervention effects.

The self-reported PA-based comparison performed during the one-year follow-up was not part of the original trial design and therefore was not powered a priori. Because this analysis was conducted post hoc, we performed an additional repeated-measures ANOVA power check (within–between interaction), assuming an effect size (ES) of f = 0.25, an α level of 0.05, statistical power of 0.80, two study groups (LAG and MVAG), and four repeated measurements. This post hoc assessment indicated that a minimum of 24 participants would provide approximately 82% statistical power for exploratory analyses. Accordingly, the PA-based comparison is presented as a secondary, exploratory analysis supported by adequate sample size but not determined through an a priori power calculation.

A complete-case analysis approach was used for all repeated-measures procedures. One participant completed the 6-month assessment (M2) but did not attend the one-year follow-up (M3). Therefore, this participant was excluded from all time-point analyses to ensure consistency in the longitudinal dataset and to avoid introducing bias associated with partial follow-up data.

Changes across the four assessments were examined using repeated-measures ANOVA. Because the primary group × time interaction did not reach statistical significance, any subsequent pairwise comparisons were conducted as exploratory post hoc contrasts, using Bonferroni-adjusted comparisons. No additional corrections across outcomes or time points were applied, consistent with the exploratory nature of these analyses.

ESs were expressed using Cohen’s *d_unbiased_* (*d_unb_*) [41] and ANOVA results were complemented with partial eta squared values (η_p_^2^). Partial eta squared was interpreted as small (0.010–0.059), medium (0.060–0.139), and large (≥0.140) [43]. Cohen’s *d_unb_* values were classified as trivial (<0.20), small (0.20–0.49), medium (0.50–0.79), or large (≥0.80) [43].

## 3. Results

### 3.1. General Characteristics

Supplementary Table S3 presents the descriptive profile of the participants included in this analysis, highlighting significant group differences in age, body mass, and BMI.

Educational level was compared between PA groups using a Chi-square test of independence. No significant differences were found between groups (χ^2^ = 1.696, *p* = 0.638), indicating that both groups had comparable educational backgrounds. A detailed distribution of educational levels is provided in Supplementary Table S4.

### 3.2. Physical and Cognitive Function

Table 1 presents the changes in physical and cognitive function across the four measurement points.

Although the group × time interaction was not statistically significant (*p* = 0.079, η_p_^2^ = 0.088), exploratory pairwise comparisons indicated that the LAG showed lower chair-stand performance than the MVAG at both the six-month (*p* = 0.027, ES = 0.76 [0.09 to 1.45]) and one-year follow-ups (*p* = 0.004, ES = 1.02 [0.34 to 1.74]). These exploratory findings do not confirm differential longitudinal effects but suggest potential directions for future investigation.

Regarding the within-group analysis, several significant differences were found. For chair-stand, both groups showed significantly higher values at post- compared to pre-intervention (LAG: *p* < 0.001, ES = 3.02 [2.02 to 4.25]; MVAG: *p* < 0.001, ES = 2.23 [1.43 to 3.25]). Similarly, values at six-month (LAG: *p* < 0.001, ES = 2.05 [1.31 to 2.95]; MVAG: *p* < 0.001, ES = 1.93 [1.22 to 2.84]), and one-year follow-ups (LAG: *p* < 0.001, ES = 2.10 [1.34 to 3.02]; MVAG: *p* < 0.001, ES = 2.09 [1.33 to 3.06]) were significantly lower than pre-intervention. The LAG also demonstrated significantly higher values at post-intervention compared to six-month (*p* < 0.001, ES = −1.03 [−1.48 to −0.66]) and one-year follow-ups (*p* < 0.001, ES = −1.24 [−1.76 to −0.81]).

In addition, for chair-stand velocity, both groups displayed significantly lower values at pre- compared to post-intervention (LAG: *p* < 0.001, ES = 2.47 [1.67 to 3.46]; MVAG: *p* < 0.001, ES = 2.16 [1.38 to 3.16]), six-month (LAG: *p* < 0.001, ES = 1.73 [1.09 to 2.49]; MVAG: *p* < 0.001, ES = 1.65 [0.93 to 2.53]), and one-year follow-ups (LAG: *p* < 0.001, ES = 1.15 [0.58 to 1.79]; MVAG: *p* < 0.001, ES = 1.38 [0.76 to 2.13]). Both groups also registered significantly lower values at one-year follow-up compared to post-intervention (LAG: *p* < 0.001, ES = −1.19 [−1.75 to −0.73]; MVAG: *p* = 0.001, ES = −0.89 [−1.40 to −0.47]). Moreover, the LAG exhibited significantly lower values at six-month follow-up compared to post-intervention (*p* = 0.001, ES = −0.55 [−0.87 to −0.28]), and at one-year follow-up than six-month follow-up (*p* < 0.001, ES = −0.59 [−0.92 to −0.29]).

Regarding TUG, both groups demonstrated significantly poor performance at pre- compared to post-intervention (LAG: *p* < 0.001, ES = −1.75 [−2.49 to −1.15]; MVAG: *p* < 0.001, ES = −1.72 [−2.57 to −1.04]), six-month (LAG: *p* < 0.001, ES = −1.57 [−2.23 to −1.04]; MVAG: *p* < 0.001, ES = −1.68 [−2.54 to −0.97]), and one-year follow-ups (LAG: *p* < 0.001, ES = −1.12 [−1.59 to −0.73]; MVAG: *p* < 0.001, ES = −1.78 [−2.69 to −1.04]). The LAG also presented significantly poorer performance at one-year than six-month follow-up (*p* = 0.015, ES = 0.28 [0.06 to 0.52]).

For SMBT, both groups showed significantly lower values at pre- compared to post-intervention (LAG: *p* < 0.001, ES = 0.69 [0.38 to 1.06]; MVAG: *p* < 0.001, ES = 0.79 [0.46 to 1.19]), and six-month follow-up (LAG: *p* = 0.001, ES = 0.45 [0.21 to 0.73]; MVAG: *p* = 0.011, ES = 0.37 [0.12 to 0.64]). Similarly, both groups demonstrated significantly higher values at post-intervention than six-month (LAG: *p* = 0.004, ES = −0.26 [−0.44 to −0.09]; MVAG: *p* < 0.001, ES = −0.41 [−0.63 to −0.22]), and one-year follow-ups (LAG: *p* < 0.001, ES = −0.41 [−0.61 to −0.24]; MVAG: *p* < 0.001, ES = −0.44 [−0.72 to −0.21]). In addition, the MVAG displayed significantly higher values at one-year follow-up compared to pre-intervention (*p* = 0.028, ES = 0.41 [0.12 to 0.73]).

For 6MWT, both groups presented significantly lower values at pre- compared to post-intervention (LAG: *p* < 0.001, ES = 0.88 [0.45 to 1.36]; MVAG: *p* < 0.001, ES = 1.11 [0.53 to 1.78]), and six-month follow-up (LAG: *p* = 0.002, ES = 0.63 [0.29 to 1.01]; MVAG: *p* = 0.004, ES = 0.81 [0.26 to 1.41]). While the LAG demonstrated significantly lower values at one-year follow-up compared to post-intervention (*p* = 0.029, ES = −0.31 [−0.57 to −0.06]), the MVAG exhibited significantly lower values at pre-intervention compared to one-year follow-up (*p* = 0.008, ES = 0.98 [0.44 to 1.61]).

For the HGS (dominant-side), both groups revealed significantly lower values at pre- compared to post-intervention (LAG: *p* < 0.001, ES = 0.71 [0.41 to 1.07]; MVAG: *p* < 0.001, ES = 0.68 [0.39 to 1.03]), six-month (LAG: *p* < 0.001, ES = 0.65 [0.39 to 0.96]; MVAG: *p* < 0.001, ES = 0.67 [0.35 to 1.05]), and one-year follow-ups (LAG: *p* < 0.001, ES = 0.66 [0.39 to 0.98]; MVAG: *p* < 0.001, ES = 0.67 [0.35 to 1.06]). Analogously, on the non-dominant side, both groups demonstrated significantly lower values at pre- compared to post-intervention (LAG: *p* < 0.001, ES = 0.62 [0.35 to 0.93]; MVAG: *p* < 0.001, ES = 0.77 [0.39 to 1.21]), six-month (LAG: *p* < 0.001, ES = 0.70 [0.43 to 1.03]; MVAG: *p* < 0.001, ES = 0.78 [0.42 to 1.22]), and one-year follow-ups (LAG: *p* < 0.001, ES = 0.56 [0.31 to 0.85]; MVAG: *p* < 0.001, ES = 0.76 [0.39 to 1.19]).

Finally, the LAG presented significantly lower values at pre- compared to post-intervention (*p* = 0.011, ES = 0.73 [0.18 to 1.34]), six-month (*p* = 0.009, ES = 0.68 [0.19 to 1.21]), and one-year follow-ups (*p* = 0.016, ES = 0.62 [0.16 to 1.13]) on the MMSE.

## 4. Discussion

The present study examined whether PA levels assessed at follow-up were associated with subsequent patterns of physical and cognitive function. Three important results should be highlighted: (i) except for SMBT and 6MWT in the LAG, every physical performance measure showed improvements at follow-up relative to pre-intervention in PA both groups; (ii) exploratory analyses suggested that higher PA levels reported during the follow-up were associated with trends toward better chair-stand performance (lower body strength), although no significant group × time interaction was found; and (iii) general cognitive function, assessed by the MMSE, decreased post-intervention in both groups, regardless of the PA level. These findings are clinically relevant, as they underscore the potential role of sustained PA in supporting independence, autonomy, and a lower risk of comorbidities within this population [44,45]. Brusa et al. [46] recently reported that higher PA levels, assessed with the IPAQ-SF, may help support spinal sagittal alignment and balance. Given the small sample size, these findings should be viewed as preliminary, and investigations with larger cohorts are needed to verify and expand upon these findings.

Rather than following earlier approaches that limited participants to their habitual PA or advised against starting structured exercise routines [6,7,8,9,10,23,24], this study promoted the continuation of higher activity levels and supported the adoption of new exercise programs throughout the follow-up phase. This approach followed the recommendation of the research committee, which emphasized promoting PA rather than limiting it [25]. Table S2 summarizes the self-reported activities performed by participants at the one-year follow-up.

Although PA was assessed using the IPAQ-SF, a commonly applied self-report tool in aging research [28,46,47], its inherent limitations must be acknowledged. As a questionnaire-based measure, it is vulnerable to recall errors, social desirability influences, and the tendency to overestimate activity levels, particularly in older populations. Even with these limitations, the IPAQ-SF continues to offer a low-burden and practical option for collecting PA data in community-based research settings [46,48]. Its feasibility and low operational demands were especially relevant in the context of a one-year follow-up, during which maintaining participant engagement and compliance can be challenging. Given these considerations, future work should aim to pair self-reported PA tools with objective monitoring approaches, including accelerometers, to strengthen accuracy and limit bias. Even so, given the scarcity of data on the durability of exercise-related adaptations and PA behaviours during extended follow-up periods, the current results provide valuable information for exercise professionals and public health practitioners by illustrating how older adults maintain PA over time and how these behaviours may relate to the retention of functional outcomes.

Despite these methodological differences, the present findings are consistent with previous RT studies reporting that improvements tend to disappear gradually during the follow-up periods [7,8,9,10,23,24]. In the current study, declines were more pronounced among participants with lower PA levels (Table 1). These reductions may be related to the absence or reduction in specific training stimuli, such as rapid force production, sustained muscle tension, and coordinated activation of agonist and antagonist muscles [22], combined with the natural progression of age-related neuromuscular deterioration, including the loss of fast-twitch fibres and a reduced pool of functional muscle units [49].

The physical function tests used in this study assess key components of daily living, including muscle strength and power, agility, dynamic balance, and aerobic capacity [34]. For the chair-stand test, the MVAG showed significantly better performance than the LAG at both the six-month (*moderate* ES) and one-year follow-ups (*large* ES). Importantly, both groups demonstrated nearly identical performance at post-intervention (M1: LAG = 24.80; MVAG = 24.63), and the divergence emerged only during the follow-up period. This pattern provides exploratory evidence, suggesting that higher self-reported PA levels assessed at the one-year follow-up may be linked to better maintenance of lower-body strength. However, given the lack of a robust statistical interaction, this divergence should be interpreted with caution as a hypothesis-generating observation. For example, while the LAG exhibited a substantial reduction from M1 to M3 (24%), the MVAG showed only a 5.6% decrease. Similar patterns have been reported in previous detraining studies in which participants were restricted in their PA levels [6,7,8,9,23]. Additionally, muscle strength assessed by HGS was preserved in both groups throughout the follow-up, with significant improvements from pre-intervention to 12 months. This result is clinically meaningful, as HGS is a well-established predictor of cause-specific mortality [50], and prolonged hospital stays [51]. As the MVAG predominantly engaged in aerobic activities during follow-up (Table S2), any mechanistic interpretation linking habitual PA to the preservation of muscle strength should be made cautiously. The observed trends likely reflect general PA-related maintenance rather than RT-specific neuromuscular adaptations, as suggested by previous research [52].

Regarding muscle power, no previous studies have monitored the chair-stand velocity, and only a few have used the SMBT to assess upper-limb power in older adults [7,23]. Fleming et al. [53] emphasized the importance of evaluating velocity during the rising phase of the chair-stand, identifying it as a key indicator for distinguishing fallers from non-fallers. The speed of the sit-to-stand movement can serve as a practical indicator of how effectively an older adult responds to everyday environmental challenges, such as uneven surfaces, rugs, or bathroom fixtures, highlighting its relevance as a fall-risk assessment tool [53]. Similarly, the SMBT is widely recognized as a useful measure of upper-limb muscle power in older adults [36]. In this study, both PA groups exhibited comparable reductions in muscle power from M2 to M3. Although performance remained above pre-intervention levels, the decline likely reflects the absence of resistance-training-specific stimuli during the follow-up period, including reduced motor unit recruitment and lower neuromuscular activation [54]. It should also be emphasised that this investigation used a heavier medicine ball (3 kg) than previous studies (2 kg [7] and 1.5 kg [23]), yet the pattern of post-intervention decline, while still above pre-intervention, was consistent with earlier findings.

Agility and dynamic balance, assessed using the TUG, have been described as highly sensitive to the adverse effects of exercise cessation [55]. While some studies have confirmed this sensitivity [8,9,10], the present results did not replicate those findings. TUG performance remained relatively stable throughout the follow-up, with the MVAG maintaining post-intervention values and the LAG showing only a modest decline (7%). The recommendation for participants to maintain an active lifestyle may help explain these results, which differ from previous detraining studies [8,9,10]. In contrast, improvements in aerobic capacity achieved through HSRT, assessed by the 6MWT, were gradually lost over the follow-up period, particularly in the LAG (5% decline vs. 1% in the MVAG). This pattern aligns with earlier research [7,8]. As shown in Table S2, MVAG participants predominantly engaged in cardiovascular activities (e.g., daily walking, hydro-gymnastics), while only one participant reported continuing structured RT, consistent with a previous study [24]. In contrast, most LAG participants performed low-intensity or sporadic activities, with several reporting no PA at all.

In this study, cognitive function, evaluated as a secondary outcome using the MMSE, is commonly applied in older populations [56]. However, it has known limitations, as scores can be affected by factors such as educational background and age, particularly when used as the sole measure [56]. Ferreira et al. [56] noted that only three studies have examined cognitive outcomes following RT interventions [11,57,58], with follow-up durations ranging from one [57] to three months [11]. In the present study, both PA groups showed small decreases in cognitive function over the one-year follow-up, although values remained slightly higher than pre-intervention. Sanchez-Lastra et al. [11], who assessed cognitive function using the MMSE after an RT program in institutionalized older adults, suggested that RT may help slow cognitive decline. A possible explanation proposed in the literature is that RT may stimulate physiological processes, such as increased production of insulin-like growth factor-1 (IGF-1), which has been linked to hippocampal neurogenesis [59], and enhanced brain myelination through oligodendrocyte development [60] that could help support cognitive function over time. We did not assess these mechanisms in this study but may help contextualize the observed patterns during the follow-up. Despite these findings, the cognitive results should be interpreted with caution. The MMSE provides a general overview of cognitive function rather than a domain-specific assessment, which represents a limitation of the study.

This study offers several advantages, including its longitudinal design, repeated objective assessments of physical function, and the classification of participants according to their habitual PA during the one-year follow-up. Nonetheless, some limitations should be considered. The first relates to the use of a self-reported instrument (IPAQ-SF) for categorising PA levels, which may introduce recall-related inaccuracies and misclassification.

Second, the observational nature of the follow-up analysis introduces the possibility of reverse causality. The IPAQ-SF reflects PA performed during the preceding seven days, meaning that the M3 measurement represents a single snapshot rather than a longitudinal indicator of habitual PA across the entire follow-up period. As such, participants who retained greater physical function may have been more capable of engaging in higher levels of PA at M3, which could contribute to the observed association. This direction-of-effect issue prevents causal inference and reinforces that the findings should be interpreted strictly as associations rather than evidence of PA-driven preservation of function. Third, the original clinical trial was registered retrospectively, as registration occurred after participant recruitment had begun. However, this delay did not affect the study design, procedures, or the validity of the results, and the protocol was fully established before data collection commenced. Fourth, although both sexes were represented, the very small number of male participants (three per group) prevented any meaningful sex-specific analyses. As a result, potential sex-related differences in long-term adaptations could not be examined and should be explored in future work with more balanced samples. Finally, because both the assessor and participants were aware of the study aims, expectancy-related influences cannot be entirely ruled out.

## 5. Clinical Applications

The preliminary findings reinforce that RT, particularly HSRT, can produce meaningful gains in physical function in older adults [32]. However, the follow-up results suggest that maintaining these gains may depend on continued engagement in PA. Consistent with previous research, muscle strength, power, and aerobic capacity declined gradually over the one-year period, with more pronounced reductions among participants reporting light PA levels. In contrast, the attenuated declines observed in the MVAG indicate that moderate-to-vigorous PA may help preserve functional capacity and slow age-related deterioration. By allowing participants to remain active during follow-up, rather than imposing PA restrictions as commonly done in detraining studies, this study provides an ecologically valid perspective on how older adults naturally integrate PA after completing a structured HSRT program. Although modest reductions in global cognition were observed, values remained slightly above pre-intervention levels, contributing to the limited evidence on cognitive trajectories following resistance training. Based on the present findings, three practical implications emerge:

1. HSRT is effective for improving lower-limb power, mobility, and upper-body strength in older adults.
2. Participants reporting higher PA levels at the one-year follow-up exhibited smaller declines in physical function, suggesting that continued engagement in PA may help sustain gains achieved during HSRT.
3. The more pronounced declines among lightly active individuals highlight the need to educate older adults about the measurable impact of reduced daily activity.

## 6. Conclusions

In conclusion, the present findings suggest that improvements in physical function achieved following the 16-week HSRT programme were largely maintained 12 months after the intervention. Based on exploratory analyses, participants who reported higher levels of self-reported PA assessed at the one-year follow-up generally demonstrated more favourable trends in functional outcomes, particularly in chair-stand performance. These observations, however, do not constitute confirmatory evidence of superior maintenance in the MVAG group due to the non-significant interaction effect. Although cognitive function declined in both groups over time, MMSE scores remained slightly above pre-intervention values.

Given the observational nature of the follow-up, the modest sample size, and the very small number of male participants, as well as the absence of a significant group-by-time interaction, the between-group differences should be viewed as exploratory rather than evidence of a causal influence of long-term PA. Overall, the results suggest that higher PA levels may relate to better preservation of functional capacity in community-dwelling older adults after HSRT, although confirmation in larger, adequately powered longitudinal studies is still required.

## Figures and Tables

**Table 1:** Changes in intervention effects based on participants’ physical activity levels at one-year follow-up.

Measures	Groups	Intervention		Follow-Up		Time Effect	Group × Time Interaction	Group Effect
		M0 Pre	M1 Post	M2 Six-Month	M3 One-Year			
Chair-stand (rep)	LAG **^a,b,c,d,e^**	12.00 ± 2.27	24.80 ± 5.29	19.55 ± 4.44 †	18.85 ± 3.80 †	F = 173.152 ¥ ***p*** **< 0.001** η_p_^2^ = 0.836 §	F = 7.346 ¥ ***p*** **= 0.001** η_p_^2^ = 0.178 §	F = 3.287 *p* = 0.079 η_p_^2^ = 0.088 *
	MVAG **^a,b,c^**	13.69 ± 4.01	24.63 ± 5.23	23.31 ± 5.35	23.25 ± 4.66			
Chair-stand velocity (m/s)	LAG **^a,b,c,d,e,f^**	0.48 ± 0.09	0.78 ± 0.13	0.70 ± 0.14	0.62 ± 0.13	F = 111.810 ¥ ***p*** **< 0.001** η_p_^2^ = 0.767 §	F = 1.384 ¥ *p* = 0.257 η_p_^2^ = 0.039 #	F = 1.567 *p* = 0.219 η_p_^2^ = 0.044 #
	MVAG **^a,b,c,e^**	0.52 ± 0.14	0.78 ± 0.10	0.73 ± 0.11	0.69 ± 0.10			
TUG (sec)	LAG **^a,b,c,f^**	6.84 ± 1.03	5.27 ± 0.65	5.35 ± 0.76	5.62 ± 1.06	F = 113.223 ¥ ***p*** **< 0.001** η_p_^2^ = 0.769 §	F = 1.303 ¥ *p* = 0.276 η_p_^2^ = 0.073 *	F = 0.903 *p* = 0.415 η_p_^2^ = 0.052 *
	MVAG **^a,b,c^**	6.59 ± 1.05	5.07 ± 0.56	5.11 ± 0.56	5.07 ± 0.47			
SMBT (m)	LAG **^a,b,d,e^**	2.05 ± 0.59	2.47 ± 0.58	2.31 ± 0.55	2.23 ± 0.55	F = 40.419 ¥ ***p*** **< 0.001** η_p_^2^ = 0.543 §	F = 0.797 ¥ *p* = 0.467 η_p_^2^ = 0.023 #	F = 0.013 *p* = 0.909 η_p_^2^ = 0.001
	MVAG **^a,b,c,d,e^**	2.04 ± 0.57	2.55 ± 0.64	2.27 ± 0.63	2.27 ± 0.51			
6MWT (m)	LAG **^a,b,e^**	473.72 ± 64.59	535.98 ± 71.68	516.73 ± 67.19	510.38 ± 86.73	F = 21.330 ¥ ***p*** **< 0.001** η_p_^2^ = 0.385 §	F = 0.556 ¥ *p* = 0.604 η_p_^2^ = 0.016 #	F = 1.816 *p* = 0.187 η_p_^2^ = 0.051 *
	MVAG **^a,b,c^**	495.26 ± 50.91	556.54 ± 54.13	540.83 ± 56.32	549.27 ± 53.42			
HGS, DS (kg)	LAG **^a,b,c^**	22.89 ± 6.37	27.69 ± 6.56	27.24 ± 6.46	27.39 ± 6.68	F = 48.862 ***p*** **< 0.001** η_p_^2^ = 0.590 §	F = 0.064 *p* = 0.979 η_p_^2^ = 0.002	F = 0.098 *p* = 0.756 η_p_^2^ = 0.003
	MVAG **^a,b,c^**	22.34 ± 5.95	27.01 ± 7.28	26.78 ± 6.95	26.53 ± 6.15			
HGS, non-DS (kg)	LAG **^a,b,c^**	21.73 ± 7.48	26.47 ± 7.24	26.79 ± 6.34	26.03 ± 7.18	F = 51.777 ¥ ***p*** **< 0.001** η_p_^2^ = 0.604 §	F = 0.103 ¥ *p* = 0.912 η_p_^2^ = 0.003	F = 0.475 *p* = 0.495 η_p_^2^ = 0.014 #
	MVAG **^a,b,c^**	20.39 ± 4.61	24.88 ± 6.36	25.08 ± 6.59	24.74 ± 6.19			
MMSE (score)	LAG **^a,b,c^**	27.20 ± 3.19	29.00 ± 0.97	28.90 ± 1.12	28.80 ± 1.39	F = 12.757 ¥ ***p*** **< 0.001** η_p_^2^ = 0.273 §	F = 0.265 ¥ *p* = 0.730 η_p_^2^ = 0.008	F = 1.184 *p* = 0.284 η_p_^2^ = 0.034 #
	MVAG	27.94 ± 1.57	29.37 ± 0.81	29.13 ± 1.09	29.19 ± 0.98			

Significant differences between periods are highlighted in bold (*p* ≤ 0.05). Abbreviations: LAG, light activity group; MVAG, moderate-to-vigorous activity group; rep, repetitions; V, velocity; m/s, meters per second; sec, seconds; m, meters; kg, kilograms; SMBT, seated medicine ball throw; 6MWT, six-minute walking test; HGS, handgrip strength test; DS, dominant side; MMSE, mini mental score examination. ¥, Greenhouse–Geisser correction. Significant differences: †, between groups at that assessment point; ^a^, pre-intervention vs. post-intervention; ^b^, pre-intervention vs. six-month follow-up; ^c^, pre-intervention vs. one-year follow-up; ^d^, post-intervention vs. six-month follow-up; ^e^, post-intervention vs. one-year follow-up; ^f^, six-month follow-up vs. one-year follow-up. η_p_^2^ values thresholds: #, small effect: 0.010 to 0.059; *, medium effect: 0.060 to 0.140; §, large effect: >0.140.

## Data Availability

The data presented in this study are available on request from the corresponding author due to ethical restrictions related to participant confidentiality and the conditions specified in the approved informed-consent procedures.

## Supplementary Materials

The following supporting information can be downloaded at: https://www.mdpi.com/article/10.3390/sports14090386/s1, Table S1: Distribution of participants according to their level of physical activity at one-year follow-up (*n* = 36).; Table S2: Distribution of self-reported physical activities at one-year follow-up: light vs. moderate-to-vigorous intensity.; Table S3: General characteristics of the sample (Mean ± SD); Table S4: Distribution of educational levels by physical activity group at one-year follow-up (number and percentage); IPAQ-SF_Scoring (Excel file).

## Abbreviations

The following abbreviations are used in this manuscript:

RT	Resistance Training
TUG	Timed up and go test
HGS	Handgrip strength test
MMSE	Mini-Mental State Examination
HSRT	High-speed resistance training
PA	Physical activity
IG	Intervention group
CG	Control group
IPAQ-SF	International Physical Activity Questionnaire
LAG	Light activity group
MVAG	Moderate-to-vigorous activity group
SMBT	Seated medicine ball throw test
6MWT	Six-minute walk test
ES	Effect Size
