# Supplementary material for: Associations of Physical Activity with One-Year Functional and Cognitive Trajectories Following High-Speed Resistance Training in Older Adults
Source: Sports (Basel). 2026 Sep 2;14(9):386. doi: 10.3390/sports14090386 (PMC13611205; doi:10.3390/sports14090386)
Supplement: Supplementary file 1 [file sports-14-00386-s001.zip › sports-4374674-supplementary-english.pdf]

Table S1: Distribution of participants according to their level of physical activity at 12-month follow-up (N=36).

Physical Activity Levels	Intervention		Follow-up	
	M0 – Pre N (%)	M1 – Post N (%)	M2 – 6-Month N (%)	M3 – 12-Month N (%)
Light	11 (30.6)	0 (0)	10 (27.8)	20 (55.6)
Moderate	25 (69.4)	4 (11.1)	22 (61.1)	11 (30.6)
Vigorous	0 (0)	32 (88.9)	4 (11.1)	5 (13.9)

Table S2: Distribution of self-reported physical activities at 12-month follow-up: light *vs.* moderate-to-vigorous intensity.

Groups	Activities self-reported
LAG	1 Participant – Occasionally walks at low intensity and yoga therapy<br>2 Participants – Once-a-week outdoor activity<br>4 Participants – Biweekly hydro gymnastics<br>6 Participants – Occasionally walks at low intensity<br>7 Participants – Did not perform any physical activity
MVAG	1 Participant – 2–3 sessions per week of moderate-intensity continuous training, high-intensity interval training, and 2–3 walks longer than 40 min<br>1 Participant – Resistance training at least 2 times per week<br>2 Participants – Walk every day and biweekly hydro gymnastics<br>5 Participants – Walks longer than 40 min every day<br>7 Participants – 2–3 sessions per week of moderate-intensity continuous training and high-intensity interval training

Abbreviations: LAG, light activity group; MVAG, moderate-to-vigorous activity group.

Table S3: General characteristics of the sample (Mean ± SD).

Measures	Groups	Intervention		Follow-up		*Group x Time Interaction*	*Group Effect*
		**M0 Pre**	**M1 Post**	**M2 6-Months**	**M3 One-year**		
Age (years)	LAG [a,b,c]	68.55 ± 3.52	69.15 ± 3.57	69.45 ± 3.52	70.00 ± 3.66	F=2.756¥ *p*=0.068 η^2_p=0.075*	F=0.013 *p*=0.908 η^2_p=0.001
	MVAG [b,c,d,e]	67.31 ± 2.06	67.50 ± 2.09	68.19 ± 2.01	68.50 ± 2.09		
Weight (kg)	LAG	67.89 ± 10.48	67.93 ± 11.37	66.64 ± 11.51	66.59 ± 11.01	F=2.522¥ *p*=0.086 η^2_p=0.069*	F=0.110 *p*=0.742 η^2_p=0.003
	MVAG [b,c,e]	68.68 ± 11.39	67.52 ± 11.90	66.32 ± 12.00	64.79 ± 11.89		
BMI (kg/m^2)	LAG	27.32 ± 4.32	27.31 ± 4.51	26.75 ± 4.47	26.68 ± 4.24	F=1.650 *p*=0.182 η^2_p=0.046#	F=0.217 *p*=0.644 η^2_p=0.006
	MVAG [b,c,e]	28.26 ± 3.57	27.76 ± 3.78	27.26 ± 3.88	26.58 ± 3.68		

Significant differences between periods are highlighted in bold ($p \leq 0.05$). Abbreviations: LAG, light activity group; MVAG, moderate-to-vigorous activity group; Kg, kilograms; BMI, body mass index; m, meters.
¥, Greenhouse-Geisser correction.
Significant differences:
a, pre-intervention *vs.* post-intervention;
b, pre-intervention *vs.* 6-month follow-up;
c, pre-intervention *vs.* one-year follow-up;
d, post-intervention *vs.* 6-month follow-up;
e, post-intervention *vs.* one-year follow-up;
η_p^2 values thresholds:
#, small effect: 0.010 to 0.059;
*, medium effect: 0.060 to 0.140;
§, large effect large: > 0.140.

Table S4: Distribution of educational levels by physical activity group at 12-month follow-up (number and percentage).

Educational Level Categories	Overall (*n*= 36)	Group		*p*-value
		LAG (*n*= 20)	MVAG (*n*= 16)	
Primary education	6 (16.7)	4 (20)	2 (12.5)	0.638[a]
Secondary education	19 (52.8)	9 (45)	10 (62.5)	
Professional education	1 (2.8)	1 (5)	0 (0)	
Higher education	10 (27.8)	6 (30)	4 (25)	

Data are presented as absolute counts and percentages. Abbreviations: LAG, light activity group; MVAG, moderate-to-vigorous activity group.

a, Values computed using the Pearson Chi-Square test.
